# New species and records of *Metriocnemus* van der Wulp s. str. from China (Diptera, Chironomidae)

**DOI:** 10.3897/zookeys.387.6408

**Published:** 2014-03-11

**Authors:** Xing Li, Xin-hua Wang

**Affiliations:** 1 College of Life Sciences, Nankai University, Tianjin 300071, China

**Keywords:** Chironomidae, *Metriocnemus*, new species, new records, China

## Abstract

The Chinese species of *Metriocnemus* van der Wulp **s. str.**, 1874 is reviewed. *M. (M.) calcaneum* **sp. n.** is described and illustrated as adult male. *M. (M.) albolineatus* (Meigen) is recorded from China for the first time. *M. (M.) beringensis* (Cranston & Oliver), *M. (M.) bilobatus* Makarchenko & Makarchenko, *M. (M.) caudigus* Sæther, *M. (M.) intergerivus* Sæther, *M. (M.) tamaokui* Sasa and *M. (M.) tristellus* Edwards are recorded from the Oriental Region for the first time. A key to the males of 17 Chinese *Metriocnemus (Metriocnemus*) species is given.

## Introduction

The genus *Metriocnemus* van der Wulp was erected in 1874; Coquillett (1910) subsequently designated *Chironomus albolineatus* Meigen, 1818 as the type species. Langton and Cobo (1997) described subgenera *Inermipupa* based on a species with a highly aberrant pupa (*Metriocnemus (Inermipupa) camencitabertarum*). Subsequently Ashe and O’Connor (2000) erected the subgenus *Crymaleomiva* for a species which not fully fits the larva, pupa or adult diagnosis of the genus (*Metriocnemus (Crymaleomvia) brunneri*).

The genus is found in mosses, phytotelmata, springs, ditches, streams and occasionally in the middle of lakes and rock pools (Cranston, Oliver and Sæther 1989, Sæther 1989).The genus presently includes 67 recorded species and had a worldwide distribution. Twelve species occur in the Oriental Region, 38 in the Palaearctic Region, 15 in the Nearctic Region, 7 in the Neotropical Region, 6 in the Afrotropical Region and one in the Australasian Region (Ashe and O’Connor 2012). Grounded on virga present or absent, Sæther (1995) divided the genus into two main groups, the *eurynotus* group (with well developed virga) and the *fuscipes* group (without virga).

Regarding taxonomic study on the genus *Metriocnemus* in China, Sæther (1995) recorded 7 species including 3 species originally described from China. Wang (2000) listed ten species of *Metriocnemus* from China, among which the species of *Metriocnemus gracei* Edwards, 1929 should be *Metriocnemus tristellus* Edwards after we re-examined the specimen. Based on new material from China, one new species is described below and seven additional species are newly recorded. A key to the males of the *Metriocnemus* s.str. species occurring in China is presented.

## Material and methods

The material examined was mounted on slides in Canada balsam, following the procedure outlined by Sæther (1969). The morphological nomenclature follows Sæther (1980). Measurements are given as ranges.

The types and other examined material in this study are housed in the College of Life Sciences, Nankai University, China (BDN).

## The species

### Metriocnemus (Metriocnemus) aculeatus

Chaudhuri & Bhattacharyay

http://species-id.net/wiki/Metriocnemus_aculeatus

Metriocnemus aculeatus Chaudhuri & Bhattacharyay in Chaudhuri et al. (1989: 309), Sæther (1995: 55), Wang (2000: 637).

#### Material examined.

CHINA: Sichuan Province, Jinfo Mountain, 30°3'30"N, 103°53'15"E, 1 male, 9.v.1986, light trap, X. Wang. Fujian Province, Wuyi Mountain, 27°43'46"N, 118°1'52"E, 4 males, 28–30.iv.1993, light trap, X. Wang. Guizhou Province, Fanjing Mountain, Huguo Temple, 27°54'43"N, 108°38'35"E, 4 males, 2–4.vii.2001, light trap, R. Zhang.

#### Remarks.

The species differs from all other *Metriocnemus* species by the wing chaetotaxy, the characteristic hypopygium with very long, sharply pointed anal point and a gonostylus lacking crista dorsalis. The Chinese specimens has higher antennal ratio (AR = 1.00–1.73) than the Indian specimens (AR = 1.09). Among the specimens of China, one was identified as *Metriocnemus hirticollis* (Staeger, 1839), which should be *Metriocnemus (Metriocnemus) albolineatus* (Meigen, 1818) after we re-examined the specimen.

#### Distribution.

*Metriocnemus (Metriocnemus) aculeatus* has been recorded from India (Chaudhuri et al. 1989) and the Oriental China.

### Metriocnemus (Metriocnemus) albolineatus

(Meigen)

http://species-id.net/wiki/Metriocnemus_albolineatus

Chironomus albolineatus Meigen, 1818: 39
Chironomus atratulus Zetterstedt, 1850: 3590
Metriocnemus albolineatus (Meigen); Sæther (1989: 399)

#### Material examined.

CHINA: Sichuan Province, Ganzi City, Yajiang County, 29°53'48"N, 103°10'19"E, 1 male, 14.vi.1986, light trap, X. Wang.

#### Remarks.

The male can be separated from other species of *Metriocnemus* by having a short virga (23–26 µm long), very weak inferior volsella, rounded to bluntly triangular crista dorsalis, tapering anal point with blunt apex, Sc with 10–31 setae, M with 15–32 setae, and cell m with 35–73 setae. This species is a member of the *eurynotus* group, but the male can be separated from other species of the group by having a shorter virga and a weaker inferior volsella. It has more setae on the wings than *Metriocnemus (Metriocnemus) brusti* and *Metriocnemus (Metriocnemus) corticalis*, but fewer than *Metriocnemus (Metriocnemus) eurynotus*.

#### Distribution.

The species is recorded from the Nearctic, Palaearctic and Oriental regions (Ashe and O’Connor 2012). In China it was collected from Sichuan Province in Oriental China for first time.

### Metriocnemus (Metriocnemus) beringensis

(Cranston & Oliver)

http://species-id.net/wiki/Metriocnemus_beringensis

Apometriocnemus beringensis Cranston & Oliver, 1988: 428
Metriocnemus beringensis (Cranston & Oliver); Sæther (1995: 59)

#### Material examined.

CHINA: Tibet, Zhangmu, 28°46'26"N, 87°30'22"E, 1 male, 15.viii.1987, light trap, C. Deng. Tibet, Zhangmu, 28°46'26"N, 87°30'22"E, 1 male, 6.ix.1987, light trap, C. Deng.

#### Remarks.

*Metriocnemus (Metriocnemus) beringiensis* may be no more than a small form of *Metriocnemus (Metriocnemus) fuscipes* (Meigen, 1818) differing in having a strongly reduced or absent anal point and slightly fewer setae on squama. Chinese specimens have fewer setae (R with 18–28, R_1_ with 18–20, R_4+5_ with 15–38 setae) on the wing than Norwegian specimens (R with 39–69, 55, R_1_ with 27–47, 36, R_4+5_ with 37–81, 59 setae) (Sæther 1995).

#### Distribution.

The species is previously recorded from the Nearctic and Palaearctic regions (Ashe and O’Connor 2012). In China it was collected in Tibet in Oriental China for first time.

### Metriocnemus (Metriocnemus) bilobatus

Makarchenko & Makarchenko

http://species-id.net/wiki/Metriocnemus_bilobatus

Metriocnemus bilobatus Makarchenko & Makarchenko, 2004: 216

#### Material examined.

CHINA: Sichuan Province, Kangding City, Ertaizidaoban, 30°2'20"N, 101°50'6"E, 1 male, 15.vi.1996, water net, X. Wang. Sichuan Province, Litang County, 29°59'45"N, 100°16'11"E, 1 male, 11.vi.1996, light trap, X. Wang.

#### Remarks.

This species can be separated from other *Metriocnemus* species by having a total length of 2.18–2.42 mm, wing length of 1.85–2.25 mm, AR = 0.23–0.26, acrostichals in 2 rows, 25–37 dorsocentrals, and reduced anal lobe of the wing. The anal point is 45–48 µm long, and lacking microtrichia in distal half. Virga is 50–53 µm long and consists of a single spine. The inferior volsella is bilobate, and the length of the basal lobe is about half the length of the gonocoxite.

#### Distribution.

The species is recorded from Russian Far East (Makarchenko and Makarchenko 2004). In China it was collected in Sichuan Province in Oriental China for first time.

### Metriocnemus (Metriocnemus) brusti

Sæther

http://species-id.net/wiki/Metriocnemus_brusti

Metriocnemus brusti Sæther, 1989: 407; Wang (2000: 637).

#### Material examined.

CHINA: Hebei Province, Pingquan County, Guangtou Mountain, 41°1'43"N, 118°43'1"E, 1 male, 29.vi.1995, light trap, H. Li. Guizhou Province, Daozhen County, Xiaosha River, 29°10'45"N, 107°32'59"E, 1 male, 25.v.2004, light trap, H. Tang.

#### Remarks.

According to Sæther (1989), the male can be separated from other members of *Metriocnemus* by having rounded crista dorsalis, anal point tapering to blunt apex, well sclerotized virga, comparatively sparsely haired wing with about 0–8 setae on Sc, 0–11 on M, 0–14 on PCu and 7–9 setae in cell m, and squama with 27–34 setae. The Chinese specimens have slightly more setae on wing membrane, with about 2–9 setae on Sc, 12–14 on M, 35–48 on PCu and 13–16 setae in cell m, but squama only with 15-17 setae. The species is similar to *Metriocnemus (Metriocnemus) albilineatus* (Meigen, 1818) and *Metriocnemus (Metriocnemus) eurynotus*, but has tapering anal point and rounded crista dorsalis. It has a well developed virga as in *Metriocnemus (Metriocnemus) corticalis* and differs from this species in minor details in the wing chaetotaxy and the hypopygium.

#### Distribution.

The species is recorded from the Nearctic and Palaearctic regions (Ashe and O’Connor 2012). It was collected in both Oriental and Palaearctic China.

### Metriocnemus (Metriocnemus) calcaneum sp. n.

http://zoobank.org/2A5C2FA0-54B9-46E6-90BA-A6D3FD4558C1

http://species-id.net/wiki/Metriocnemus_calcaneum

Figs 1–3

#### Type material.

Holotype male (BDN No. 08832), CHINA: Hebei Province, Pingquan County, Guangtou Mountain, 41°1'43"N, 118°43'1"E, 29.vi.1995, light trap, W. Bu. Paratype male (BDN No. 08816), as holotype except the date is 28.vi.1995.

#### Diagnostic characters.

The gonostylus is 83–90 µm long, with short, strong outer projection; crista dorsalis is strong and pointed; LR_1_ is high (0.68–0.70); squama with 8–10 setae; and cell m basally of RM with 15 setae.

#### Etymology.

From Latin, *calcaneum* meaning the heel, referring to the short, strong outer projection on gonostylus. The species epithet is a noun in apposition.

#### Description.

Male (n = 2).

Total length 2.49–2.68 mm. Wing length 1.68–1.88 mm. Total length / wing length 1.43–1.48. Wing length / length of profemur 2.09–2.23.

*Coloration*. Head and abdomen brown, thorax and legs yellow.

*Head*. AR 1.43–1.58. Temporal setae 11, including 4 inner verticals, 3 outer verticals, and 4 postorbitals. Clypeus with 10–12 setae. Tentorium 123–150 µm long, 18–20 µm wide. Stipes 105–108 µm long, 25–40 µm wide. Palp segments lengths (in µm): 18–40, 43, 135–155, 105–118, 153–165. Length ratio of palpomere 5/3 1.06–1.13.

**Figures 1–3.**
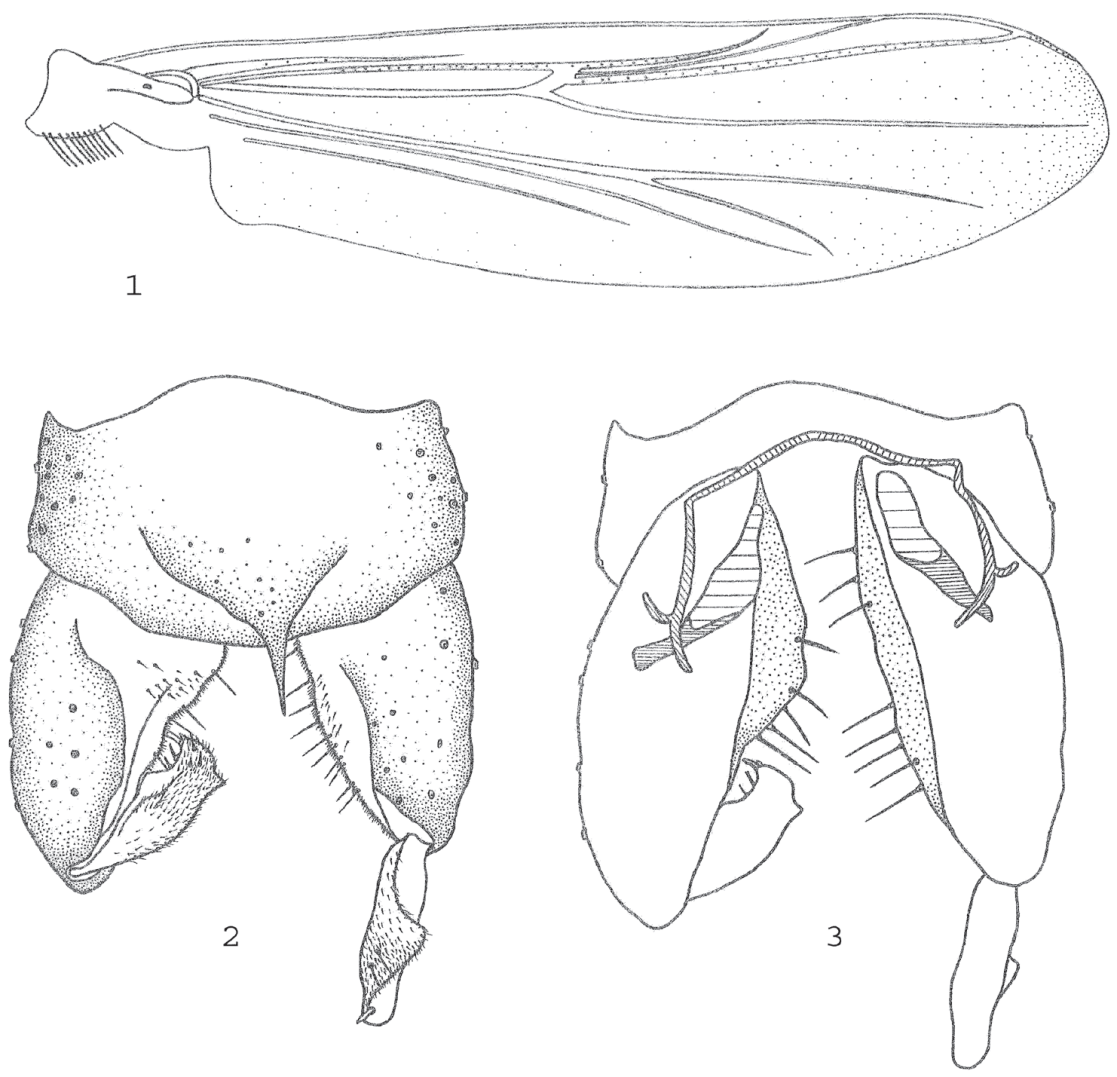
*Metriocnemus (Metriocnemus) calcaneum* sp. n., male. **1** wing **2** hypopygium (dorsal view) **3** hypopygium (ventral view).

*Wing* (Fig. 1). VR 1.16–1.34. Costal extension 110–150 µm long. Brachiolum with 1 seta, C extension with 8 non–marginal setae, Sc with 2–3, R with 18–19, R_1_ with 16–17, R_4+5_ with 25–32, RM with 1, M with 0–2, M_1+2_ with 26, M_3+4_ with 20, Cu with 0, Cu_1_ with 7 setae. Pcu and An without setae. Wing membrane with setae in most cells, with 15 setae in cell m basally of RM, cell r_4+5_ with 74–105, cell m_1+2_ with 108–135, cell m_3+4_ with 21–38 setae. Squama with 8–10 setae.

*Thorax*. Antepronotum with 1–2 setae. Dorsocentrals 10–12, acrostichals 8–9, prealars 5. Scutellum with 7–8 setae.

*Legs*. Spur of fore tibia 53–75 µm long, spurs of mid tibia 33–35 µm and 25–28 µm long, of hind tibia 53–58 µm and 28 µm long. Width at apex of fore tibia 38–43 µm, of mid tibia 35–43 µm, of hind tibia 45–50 µm. Comb of 12 setae, shortest 20–23 µm long, longest 50–63 µm long. First tarsomere of mid leg with 1 pseudospur, 20 µm long; hind leg without pseudospurs. Lengths (in µm) and proportions of legs as in Table 1.

**Table 1.** Lengths (in µm) and proportions of legs segments of *Metriocnemus (Metriocnemus) calcaneum* sp. n., male (n = 2).

	fe	ti	ta_1_	ta_2_	ta_3_	ta_4_
p_1_	800–840	830–950	580–650	310–370	198–224	136–150
p_2_	790–890	750–860	400–430	185–198	150–155	108
p_3_	840–970	920–1060	520–570	242–264	185–220	114–123
	**ta_5_**	**LR**	**BV**	**SV**	**BR**	
p_1_	98–105	0.68–0.70	2.87–2.98	2.75–2.81	2.0	
p_2_	88–98	0.50–0.53	3.63–3.94	3.85–4.07	2.44–3.3	
p_3_	93–98	0.54–0.57	3.60–3.69	3.38–56	2.91–4.0	

*Hypopygium* (Figs 2–3). Anal point proper 30–45 µm long, 23–25 µm wide, tapering from base to pointed apex; with 8–11 setae at base on tergite IX. Laterosternite IX with 10–13 setae. Phallapodeme 48–53 µm long; transverse sternapodeme 110–133 µm long. Gonocoxite 223–224 µm long. Gonostylus 83–90 µm long, with short, strong outer projection; crista dorsalis strong and pointed; megaseta 13 µm long. HR 2.49–2.70. HV 2.76–3.24.

#### Distribution.

The specimens were collected in Hebei Province in Palaearctic China.

### Metriocnemus (Metriocnemus) calvescens

Sæther

http://species-id.net/wiki/Metriocnemus_calvescens

Metriocnemus calvescens Sæther, 1995: 45; Wang (2000: 637).

#### Material examined.

CHINA: Qinghai Province, Menyuan County, Haibei Station, 37°22'33"N, 101°37'20"E, 3 males, 12.vii.1989, light trap, M. Wei.

#### Remarks.

This species apparently forms the sister species of *Metriocnemus ursinus* (Holmgren, 1869). It can be separated from *Metriocnemus (Metriocnemus) ursinus* on its smaller size, by having only 27 setae in cell r_4+5_, only 26 setae on scutellum, and the presence of pseudospurs on fore metatarsus (Sæther 1995). Compare to the holotype, the measurements of the two more males are very similar, except only 8-15 setae on scutellum.

#### Distribution.

The species was described by Sæther (1995) in Qinghai Province in Palaearctic China based on a single male specimen. Later two more male slides have been made which collected in the same location as the holotype.

### Metriocnemus (Metriocnemus) caudigus

Sæther

http://species-id.net/wiki/Metriocnemus_caudigus

Metriocnemus caudigus Sæther, 1995: 52; Wang (2000: 367).

#### Material examined.

CHINA: Fujian Province, Wuyi Mountain, 27°43'46"N, 118°1'52"E, 1 male, 28.iv.1993, light trap, X. Wang.

#### Remarks.

This species can be separated from other species of *Metriocnemus* by having a slender gonostylus with long, low crista dorsalis; a robust, long anal point; and an AR of 1.0–1.5. The Chinese specimen has a slightly higher 5^th^/3^rd^ palp ratio (0.79–0.94) than the Norwegian specimens (0.70). Further, the Chinese specimen has 2 pseudospurs, 24–32 µm long, on tarsomere 1 and 1 pseudospur, 20–28 µm long on tarsomere 2 of mid and hind legs, while the Norwegian specimens have 1 pseudospur, 23–38 µm long, on tarsomere 1 of mid and hind legs only.

#### Distribution.

The species was described from Norway by Sæther (1995). In China it was collected in Fujian Province in Oriental China.

### Metriocnemus (Metriocnemus) dentipalpus

Sæther

http://species-id.net/wiki/Metriocnemus_dentipalpus

Metriocnemus dentipalpus Sæther, 1995: 44

#### Material examined.

Holotype (ZMB No.145): CHINA: Tibet, Dingri, Chang Street, 28°39'31"N, 87°7'34"E, 1 male, 16.ix.1987, light trap, C. Deng.

#### Remarks.

The species is similar to *Metriocnemus (Metriocnemus) brusti* Sæther, 1989 and *Metriocnemus (Metriocnemus) acutus* Sæther, 1995 but can be separated on having 20 setae on vein M, an AR = 1.43, palp with a small apical tooth on each of palpomeres 3 and 4, anal point with concave margins and details of the hypopygium. *Metriocnemus (Metriocnemus) brusti* only have 0–14 setae and *Metriocnemus (Metriocnemus) acutus* without setae on vein M.

#### Distribution.

The species is known only from Tibet in Oriental China by Sæther (1995).

### Metriocnemus (Metriocnemus) eurynotus

(Holmgren)

http://species-id.net/wiki/Metriocnemus_eurynotus

Chironomus obscuripes Holmgren, 1869: 8, preoccupied
Chironomus eurynotus Holmgren, 1883: 179
Metriocnemus eurynotus (Holmgren); Sæther (1995: 44); Wang (2000: 637)

#### Material examined.

CHINA: Gansu Province, Yuzhong County, 35°50'35"N, 104°6'45"E, 1 male, 8.viii.1993, light trap, X. Wang. Sichuan Province, Jinfo Mountain, 30°3'30"N, 103°53'15"E, 1 male, 9.v.1986, light trap, X. Wang.

#### Remarks.

The male can be recognized on the completely parallel–sided anal point with broad, rounded apex; and the sharply triangular crista dorsalis.

#### Distribution.

The species is recorded from the Nearctic, Palaearctic and Oriental regions (Ashe and O’Connor 2012). The species has been collected in both Oriental and Palaearctic China.

### Metriocnemus (Metriocnemus) fuscipes

(Meigen)

http://species-id.net/wiki/Metriocnemus_fuscipes

Chironomus fuscipes Meigen, 1818: 49
Metriocnemus fuscipes (Meigen); van der Wulp (1875: 136); Sæther (1989: 423, 1995: 58); Wang (2000: 637)

#### Material examined.

CHINA: Ningxia Hui Autonomous Region, Liupan Mountain, 35°47'22"N, 106°17'36"E, 1 male, 4.viii.1987, light trap, W. Wang. Liaoning Province, Changbai Mountain, 41°2'40"N, 122°37'38"E, 1 male, 1.v.1994, light trap, X. Wang. Tibet, Yadong County, 27°29'5"N, 88°54'25"E, 1male, 29.viii.2003, light trap, H. Xue.

#### Remarks.

This species is easily recognized by the lack of virga, pointed anal point, low inferior volsella with apical hump, LR_3_ only 0.38–0.39 and AR = 0.9–1.2 (Sæther 1989). The Chinese specimens have a slightly higher AR (1.28–1.42) than that given by Sæther (1989) (AR = 0.86–1.17).

#### Distribution.

The species is recorded from the Nearctic and Palaearctic regions (Ashe and O’Connor 2012). In China the species was collected in Palaearctic China.

### Metriocnemus (Metriocnemus) intergerivus

Sæther

http://species-id.net/wiki/Metriocnemus_intergerivus

Metriocnemus intergerivus Sæther, 1995: 52

#### Material examined.

CHINA: Hubei Province, Enshi Tujia and Miao Autonomous Prefecture, He Mountain, Fenshuiling, 29°53'24"N, 110°2'1"E, 2 males, 12–16.vii.1999, light trap, B. Ji. Hubei Province, Enshi Tujia and Miao Autonomous Prefecture, Xianfeng County, Pingba, 29°24'57"N, 109°8'18"E, 2 males, 20.vii.1999, light trap, B. Ji. Liaoning Province, Changbai Mountain, 41°2'40"N, 122°37'38"E, 2 males, 1.v.1994, light trap, X. Wang. Sichuan Province, Daocheng County, Sangdui, 29°11'28"N, 100°6'32"E, 1 male, 13.vi.1996, light trap, X. Wang.

#### Remarks.

The male imago combines a well developed virga with a low crista dorsalis, weak anal point, and low inferior volsella typical of the *fuscipes* group. The species has an AR = 1.78–2.42.

#### Distribution.

The species is recorded from the Nearctic and Palaearctic regions (Ashe and O’Connor 2012). It is known from both Oriental and Palaearctic China for first time.

### Metriocnemus (Metriocnemus) picipes

(Meigen)

http://species-id.net/wiki/Metriocnemus_picipes

Chiromomus picipes Meigen, 1818
Chironomus paganicus Walker, 1856: 183
Metriocnemus hirtipalpis Kieffer, 1915: 478
Metriocnemus longipalpus Sinharay & Chaudhuri, 1978: 281
Metriocnemus picipes (Meigen); van der Wulp (1874: 136); Edwards (1929: 311); Pinder (1978: 90), Sæther (1995: 59); Wang (2000: 637)

#### Material examined.

CHINA: Jilin Province, Changbai Mountain, 41°2'40"N, 122°37'38"E, 1 male, 7.vii.1986, light trap, X. Wang. Yunan Province, Songhua County, 25°11'38"N, 109°8'18"E, 1 male, 1.vi.1996, light trap, B. Wang. Hebei Province, Pingquan County, Guangtou Mountain, 41°1'43"N, 118°43'1"E, 1 male, 29.vi.1995, light trap, H. Li. Sichuan Province, Daocheng County, Sangdui, 29°11'28"N, 100°6'32"E, 2 males, 13.vi.1996, light trap, X. Wang. Hubei Province, Deduo Mountain, Fenshui ling, 29°40'8"N, 109°5'12"E, 1 male, 16.vi.1999, light trap, B. Ji.

#### Remarks.

The Chinese specimens have a low number of setae on each of subcosta (0–9, 2) and M (4–20, 12), combined with a high AR (1.89–2.90, 2.24) and LR_3_ (0.44–0.50, 0.46), and a conspicuously long spur of the fore tibia (2.0–2.4 times as long as the apical width of tibia), these characters will distinguish the species from other members of *Metriocnemus*. *Metriocnemus wittei* Freeman, 1955 from Africa might be a synonym (Sæther 1995).

#### Distribution.

The species is recorded from the Nearctic, Palaearctic and Oriental regions (Ashe and O’Connor 2012). It has been collected in both Oriental and Palaearctic China.

### Metriocnemus (Metriocnemus) tamaokui

Sasa

http://species-id.net/wiki/Metriocnemus_tamaokui

Metriocnemus tamaokui Sasa, 1983: 77

#### Material examined.

CHINA: Sichuan Province, Daocheng City, Sangdui County, 29°11'28"N, 100°6'32"E, 1 male, 11.vi.1996, sweep net, X. Wang. Yunnan Province, Dali County, Yinqiao, 25°45'10"N, 109°5'12"E, 1 male, 21.v.1996, light trap, B. Wang. Xinjiang Uygur Autonomous Region, Yining City, Saimuli Lake, 44°37'26"N, 81°12'28"E, 1 male, 30.vii.2000, light, trap, N. Tang.

#### Remarks.

The species has an AR = 1.13; very long palp; wing with numerous macrotrichia; ta_1_ and ta_2_ of mid and hind leg each with 2 pseudospurs; virga composed of nearly 20 spines; anal point robust, partly parallel–sided; a strongly projecting, rectangular inferior volsella in basal half of the gonocoxite; and a triangular, preapical crista dorsalis.

#### Distribution.

The species was described from Japan (Sasa 1983), and it is a newly recorded in China. It is known from both Oriental and Palaearctic China.

### Metriocnemus (Metriocnemus) tristellus

Edwards

http://species-id.net/wiki/Metriocnemus_tristellus

Metriocnemus tristellus Edwards, 1929: 312
Metriocnemus gracei Edwards, 1929: 312 sensu Wang 2000: 637.

#### Material examined.

CHINA: Zhejiang Province, Qingyuan City, Baishanzu County, 27°43'51"N, 100°7'33"E, 2 males, 18–22.iv.1994, light trap, H. Wu.

#### Remarks.

The species has an AR = 1.2, very low inferior volsella, and weak anal point. Basal half of the wing membrane is bare, costa is strongly produced, and the distance C–M is rather less than M–Cu. The palp is unusually short, palpomeres 3 and 4 are less than three times as long as broad, palpomere 2 is rather longer. Wang (2000) recorded *Metriocnemus gracei* Edwards, 1929 from China, which should be *Metriocnemus (Metriocnemus) tristellus* Edwards after we re-examined the specimen.

#### Distribution.

The species is recorded from the Nearctic and Palaearctic regions (Ashe and O’Connor 2012).The species has been collected in Oriental China for first time.

### Metriocnemus (Metriocnemus) unilinearis

Chaudhuri & Bhattacharyay

http://species-id.net/wiki/Metriocnemus_unilinearis

Metriocnemus unilinearis Chaudhuri & Bhattacharyay in Chaudhuri et al. 1989: 312; Sæther (1995: 56); Wang (2000: 637)

#### Material examined.

CHINA: Tibet, Zhangmu, 28°46'26"N, 87°30'22"E, 1 male, 7.ix.1987, light trap, C. Deng. Tibet, Zhangmu, 28°46'26"N, 87°30'22"E, 1 male, 21.ix.1987, light trap, C. Deng. Tibet, Zhangmu, 28°46'26"N, 87°30'22"E, 1 male, 9.viii.1987, light trap, C. Deng. Guizhou Province, Fanjing Mountain, Huguo Temple, 27°54'43"N, 108°38'35"E, 1 male, 4.viii.2001, light trap, R. Zhang. Fujian Province, Wuyi Mountain, Tongmu County, 27 °43’ 46"N, 118°1'52"E, 2 males, 29.iv.1993, sweep net, W. Bu.

#### Remarks.

This species can be separated from other *Metriocnemus* species by the following combination of characters: acrostichals 4–5, uniserial; scutellum with 12 irregularly arranged setae; squama with 7 setae; anal point narrow and pointed with 14 setae at base, and gonocoxite with a small, dorsal, flap–like setose lobe. The specimens from Tibet have a lower antennal ratio (AR = 0.66–0.88) than the Indian specimens (AR = 1.03).

#### Distribution.

This species was described from India (Chaudhuri et al. 1989). It has been collected in both Oriental and Palaearctic China.

### Metriocnemus (Metriocnemus) wangi

Sæther

http://species-id.net/wiki/Metriocnemus_wangi

Metriocnemus wangi Sæther, 1995: 41; Wang (2000: 637)

#### Material examined.

CHINA: Sichuan Province, Jinfo Mountain, 30°3'30"N, 103°53'15"E, 2 males [holotype / paratype], 10.v.1986, light trap, X. Wang.

#### Remarks.

We examined the holotype and paratype specimens which are described by Sæther (1995) from China. According to Sæther (1995), the male can be separated from all other members of the *euryotus* group except *Metriocnemus (Metriocnemus) albolineatus* by the short virga (about 26 µm long), subcosta with 42–70 setae, and cell m with 113–144 setae. The species is close to *Metriocnemus (Metriocnemus) albolineatus* sharing the short virga and general wing chaetotaxy. It differs from *Metriocnemus (Metriocnemus) albolineatus* by having an AR = 0.5–0.6 and a much shorter anal point.

#### Distribution.

This species was only described from Oriental China by Sæther (1995).

### Key to adult males of *Metriocnemus* s. str. in China

**Table:** 

1	Gonostylus with short, strong outer projection	*Metriocnemus (Metriocnemus) calcaneum* sp. n.
–	Gonostylus without strong outer projection	2
2	Basal half of wing membrane bare or at most with scattered setae in anal cell	3
–	Entire wing membrane, except sometimes cell m basally of RM, densely clothed with setae	6
3	AR = 0.23–0.26; virga consists of 1 spine	*Metriocnemus (Metriocnemus) bilobatus* Makarchenko & Makarchenko
–	AR = 0.84–2.71; without virga or virga consists of about 10 spines	4
4	Anal point weak, tapering to a point; a few setae present in anal cell of wing; crista dorsalis low or absent, squama with about 4–11 setae	5
–	Anal point robust, rounded apically; basal half of wing membrane bare; crista dorsalis blutly triangular; squama with 27 setae	*Metriocnemus (Metriocnemus) calvescens* Sæther
5	Inferior volsella very low; without virga	*Metriocnemus (Metriocnemus) tristellus* Edwards
–	Inferior volsella distinct; virga present	*Metriocnemus (Metriocnemus) aculeatus* Chaudhuri & Bhattacharyay
6	Virga consisting of 6–14 spines; crista dorsalis preapical and triangular or occasionally long and low or gonostylus with strong preapical projection	7
–	Virga absent, crista dorsalis long and low	14
7	Crista dorsalis long and low	8
–	Crista dorsalis preapical, triangular, pointed to rounded	9
8	Anal point robust, rounded at apex, 49–68 µm long; AR about 1.3; squama with 18–23 setae	*Metriocnemus (Metriocnemus) caudigus* Sæther
–	Anal point weak, pointed, 15–26 µm long; AR = 2.0–2.4; squama with 43–64 setae	*Metriocnemus (Metriocnemus) intergerivus* Sæther
9	Inferior volsella in basal half of gonocoxite, rectangular, strongly projecting	*Metriocnemus (Metriocnemus) tamaokui* Sasa
–	Inferior vosella in basal 0.58–0.80 of gonocoxite, widest in basal half, weak to pronounced	10
10	Subcosta with 0–8 setae, cell m basally of RM with 0–29 setae	11
–	Subcosta with 10–55 setae, cell m basally of RM with 40–144 setae	12
11	Vein M with 0–11 setae, third and fourth palpomere without small, sclerotized apical tooth; anal point triangular	*Metriocnemus (Metriocnemus) brusti* Sæther
–	Vein M with about 20 setae, third and fourth palpomere with small, sclerotized apical tooth; anal point with concave margins	*Metriocnemus (Metriocnemus) dentipalpus* Sæther
12	Spines of virga 23–26 µm long, crista dorsalis rounded to bluntly triangular	13
–	Spines of virga 34–68 µm long, crista dorsalis either sharply triangular or rounded	*Metriocnemus (Metriocnemus) eurynotus* (Holmgren)
13	AR = 0.5–0.6, anal point 24–28 µm long	*Metriocnemus (Metriocnemus) wangi* Sæther
–	AR = 1.2–1.5, anal point 51–64 µm long	*Metriocnemus (Metriocnemus) albolineatus* (Meigen)
14	LR_1_ = 0.55–0.67, LR_3_ = 0.37–0.49, squama with 19–46 setae	15
–	LR_1_ about 0.66–0.88, LR_3_ about 0.54–0.59, squama with 7–10 setae	*Metriocnemus (Metriocnemus) unilinearis* Chaudhuri & Bhattacharyay
15	AR = 0.70–1.42; pseudospurs often absent on ta_2_ of hind leg	16
–	AR = 1.89–2.49; pseudospurs always present on ta_2_ of hind leg	*Metriocnemus (Metriocnemus) picipes* (Meigen)
16	Anal point absent or at most 15 µm long; LR_1_ = 0.81–0.84	*Metriocnemus (Metriocnemus) beringensis* (Cranston & Oliver)
–	Anal point strong, 43–53 µm long; LR1 = 0.57–0.60	*Metriocnemus (Metriocnemus) fuscipes* (Meigen)

## Supplementary Material

XML Treatment for Metriocnemus (Metriocnemus) aculeatus

XML Treatment for Metriocnemus (Metriocnemus) albolineatus

XML Treatment for Metriocnemus (Metriocnemus) beringensis

XML Treatment for Metriocnemus (Metriocnemus) bilobatus

XML Treatment for Metriocnemus (Metriocnemus) brusti

XML Treatment for Metriocnemus (Metriocnemus) calcaneum

XML Treatment for Metriocnemus (Metriocnemus) calvescens

XML Treatment for Metriocnemus (Metriocnemus) caudigus

XML Treatment for Metriocnemus (Metriocnemus) dentipalpus

XML Treatment for Metriocnemus (Metriocnemus) eurynotus

XML Treatment for Metriocnemus (Metriocnemus) fuscipes

XML Treatment for Metriocnemus (Metriocnemus) intergerivus

XML Treatment for Metriocnemus (Metriocnemus) picipes

XML Treatment for Metriocnemus (Metriocnemus) tamaokui

XML Treatment for Metriocnemus (Metriocnemus) tristellus

XML Treatment for Metriocnemus (Metriocnemus) unilinearis

XML Treatment for Metriocnemus (Metriocnemus) wangi
